# Proteomic Approach for Extracting Cytoplasmic Proteins from *Streptococcus sanguinis* using Mass Spectrometry

**DOI:** 10.5539/jmbr.v7n1p50

**Published:** 2017

**Authors:** Fadi El-Rami, Kristina Nelson, Ping Xu

**Affiliations:** 1 Philips Institute for Oral Health Research, Virginia Commonwealth University, Richmond, Virginia, USA; 2 Department of Microbiology and Immunology, Virginia Commonwealth University, Richmond, Virginia, USA; 3 Chemical and Proteomic Mass Spectrometry Core Facility, Department of Chemistry, Virginia Commonwealth University, Richmond, Virginia, USA

**Keywords:** proteomics, *Streptococcus sanguinis* SK36, mass spectrometry, oral cavity, soluble protein

## Abstract

*Streptococcus sanguinis* is a commensal and early colonizer of oral cavity as well as an opportunistic pathogen of infectious endocarditis. Extracting the soluble proteome of this bacterium provides deep insights about the physiological dynamic changes under different growth and stress conditions, thus defining “proteomic signatures” as targets for therapeutic intervention. In this protocol, we describe an experimentally verified approach to extract maximal cytoplasmic proteins from *Streptococcus sanguinis* SK36 strain. A combination of procedures was adopted that broke the thick cell wall barrier and minimized denaturation of the intracellular proteome, using optimized buffers and a sonication step. Extracted proteome was quantitated using Pierce BCA Protein Quantitation assay and protein bands were macroscopically assessed by Coomassie Blue staining. Finally, a high resolution detection of the extracted proteins was conducted through Synapt G2Si mass spectrometer, followed by label-free relative quantification via Progenesis QI. In conclusion, this pipeline for proteomic extraction and analysis of soluble proteins provides a fundamental tool in deciphering the biological complexity of *Streptococcus sanguinis*.

## 1. Introduction

Understanding bacterial gene functions necessitates dissecting its transcriptomic and proteomic profiles at given conditions (Wang et al., 2010). With the high pace advances in transcriptomics that govern global coverage of genome to measure its mRNA transcript counts even in a single cell, the need for efficient proteomic approaches that cover a maximal number of proteins is overwhelmingly huge to address an ever-growing list of biological questions. Any proteomic approach is a multi-step process challenged by many pitfalls at every stage, from protein extraction to high resolution quantification and extensive data analysis (Han, Aslanian, Yates, 2008; Chandramouli, Qian, 2009). Adding to the complexity of the situation is the diversity of techniques adopted by researchers for every step which impacts the sharing and comparison of results (Perez-Riverol, Alpi, Wang, Hermjakob, & Vizcaíno, 2015; Vaudel et al., 2016). As proteomic approaches for insoluble membrane proteins have been discussed elsewhere (Smith, 2011; Moore, Hess, Jorgenson, 2016), here we provide a proteomic approach for quantifying the soluble proteins in an oral commensal (Siqueira & Rôças, 2017) and opportunistic causative agent of infective endocarditis, *Streptococcus sanguinis* SK36 (Do et al., 2011; Kim et al., 2016). After sequencing the genome (Xu et al., 2007) and identifying the essential genes (Xu et al., 2011) of *S. sanguinis* SK36, the current challenge is to identify the dynamics of its proteins, especially the essential proteins, under different stress conditions that mimic clinical situations it induces, to define “pathogenesis signatures” as promising therapeutic targets.

## 2. Materials and Methods

### 2.1 Bacterial Strain

*S. sanguinis* SK36 strain was routinely grown in brain heart infusion (BHI) broth (BD, San Jose, CA) under micro-aerobic conditions (7.2% H2, 7.2% CO2, 79.6% N2, and 6% O2) at 37°C.

### 2.2 Reagents and Buffers

All buffers and solutions were prepared using ultrapure water and analytical grade reagents. All prepared reagents were stored at room temperature unless indicated otherwise. Protease Inhibitor Cocktail Set II (Calbiochem, EMD Millipore, cat # 539132) was prepared as a stock solution by adding to each vial of lyophilized protease inhibitor cocktail 1 ml of DMSO first then add 4 ml of ultrapure water. Stock solution stored at −20 °C. DL-Dithiothreitol (Sigma, cat # D9779 SIGMA) was prepared as 1 M stock solution and stored at 4 °C. Incomplete lysis buffer was prepared as follows: 50 mM Tris (pH 7.4), 150 mM NaCl, SDS 0.1% (w/v). Before usage directly, 1 ml of complete lysis buffer for each sample was prepared by mixing 100 μL of reconstituted protease inhibitors solution, 1 μL of 1 M DTT (stock), and 900 μL incomplete lysis buffer. The complete lysis buffer was stored on ice.

### 2.3 Protein Extraction

Frozen bacteria (glycerol stock at −80 °C) were inoculated into 3 ml BHI and incubated at 37°C overnight in anoxomat jars adjusted to microaerophilic conditions (6% O_2_, 7.2% CO_2_, 7.2% H_2_, and 79.6% N_2_). 400 μL of overnight grown bacteria were added into 40 ml BHI and incubated 5–5.5 hours until late log phase at the OD_600_ reading was ~ 0.8. After centrifugation, the pellet was mixed with 1 ml of freshly prepared complete lysis buffer and incubated on ice for 30 minutes. Afterwards, samples were sonicated as follows: Amplitude 35%, 5 sec ON, 10 sec OFF, for a total sonication time of 1 minute. The sonication efficiency was measured by detecting the absorbance of solution at 260 nm (A_260_) and quantifying the released DNA from the lysed cells. Tubes were centrifuged at 13,000 rpm for 15 minutes at 4°C and supernatants were stored at −80 °C or moved directly to protein quantification.

### 2.4 Protein Quantification using Pierce BCA Protein Assay Kit

Protein was quantified using Pierce BCA Protein Assay Kit (Thermo Scientific, cat # 23227) as recommended by the manufacturer. Briefly, the BCA Working Reagent (WR) was prepared by mixing 50 parts of BCA Reagent A with 1 part of BCA Reagent B (Reagent A:B ratio = 50:1). 25μL of each BCA Standard or sample was pipetted into a microplate well (Greiner Bio-one, cat # 655090) and then 200μL of the WR was added to each well. Plate was placed on a plate shaker for 30 seconds and incubated at 37°C for 30 minutes. The absorbance of each sample was measured at 562nm on a plate reader (Synergy H1 Hybrid, BioTek, United States).

### 2.5 Protein Visualization using Coomassie Blue Staining

To determine whether the soluble proteins quantified encompass proteins of various molecular weights, especially the low molecular weight proteins or less abundant proteins which were eradicated in harsh procedures, protein visualization was conducted using Coomassie Blue staining. 50 μL of each soluble protein was mixed with 50 μL of 2x Laemmli sample buffer (Biorad, cat # 161-0737) and heated for 10 min at 100 °C. Samples were stored at −80 °C or kept on ice while working. SDS-polyacrylamide gel 12% was prepared as described by Harlow and Lane (Harlow & Lane, 1988). 20 μg of every sample’s protein was loaded in a well. The gel was allowed to run for 10 minutes at 100 V then the voltage was raised to 200 V for 20–30 minutes. Gels were fixed by submerging in fixing solution (50% water; 40% Ethanol; 10% acetic acid) for 10–30 minutes, then washed in distilled water for 10 minutes, and finally stained with Coomassie Brilliant Blue R-250 staining solution (Biorad, cat # 161-0436) for 1–4 hours. To enhance visualization of bands, the gels were de-stained with de-staining solution (50% methanol, 5% acetic acid, 45% water) for 45 minutes.

### 2.6 Sample Preparation for Quantitative Mass Spectrometry

Four volumes of cold (−20°C) acetone were added to each protein sample and incubated for 60 minutes at −20°C. After centrifugation for 10 minutes at 13,000 ×g, supernatant was decanted and the acetone was allowed to evaporate from the uncapped tube at room temperature for 30 minutes. RapiGest SF working solution was reconstituted by adding 1 mg RapiGest SF powder in 50 mM Ammonium Bicarbonate (NH_4_HCO_3_) to achieve a 0.1% (w/v) solution. Every protein pellet was resuspended in 100 μL RapiGest SF working solution and vortexed thoroughly to dissolve the protein pellet. Samples were reduced with 4 μL of 10 mM dithiothreitol (DTT) in 0.1 M ammonium bicarbonate at room temperature for 30 minutes, then the samples were alkylated with 4 μL 50 mM iodoacetamide in 0.1 M ammonium bicarbonate at room temperature for 30 minutes. Finally, samples were digested with 1 μg trypsin overnight and then quenched with 5% (v:v) glacial acetic acid.

### 2.7 Proteomic Analysis by Quantitative Mass Spectrometry

Samples were analyzed by a Waters Synapt G2Si mass spectrometer system with a nanospray ion source interfaced to a Waters M-Class C18 reversed-phase capillary column. MSE scout runs were performed on each sample with spiked internal standards to determine the amount of protein on column. The injection volume was adjusted to achieve 200 ng protein on column for each analysis using ion mobility separation. Each sample was run in triplicate using this technique.

The peptides were injected onto the trap and analytical columns, and then eluted from the column by an acetonitrile/0.1% formic acid gradient at a flow rate of 0.4 μL/min over 60 minutes. The nanospray ion source was operated at 3.5 kV. A lockspray compound was used to improve the mass accuracy of the analysis. The digests were analyzed using the double play capability of the instrument acquiring full scan mass spectra at low collision energy to determine peptide molecular weights and product ion spectra at high collision energy to determine amino acid sequence. Ion mobility mode was used to produce a third dimension of separation, to maximize the number of peptide identifications. The data was analyzed by database searching using the PLGS search algorithm against Uniprot’s *Streptococcus sanguinis* database. Relative quantification was performed using Progenesis QI.

### 2.8 High Resolution Relative Quantification of Soluble Proteome Using Progenesis QI Software

Progenesis QI software (http://www.nonlinear.com/progenesis/qi-for-proteomics/download/) was used to analyze data imported with file format (.raw) from Synapt G2Si Mass Spectrometer. Each run in the experiment was shown as an ion intensity map which was representative of the sample’s MS signal by m/z and retention time. To combine and compare results from different runs, Progenesis QI aligned them to compensate for between-run variation in the chromatography. To ensure consistent peak picking and matching across all data files, we created an aggregate data set from the aligned runs.

The results of quantification and identification were automatically brought together as “compound results”. After detection, the ion abundance measurements were normalized so comparisons can be made between the runs and find compounds of biological interest. Peptides were selected based on the significance measures e.g. Anova p-value, fold change, power. Ion intensity maps, 3D views, mass spectra and chromatograms were displayed for each compound ion on all runs to provide quality assurance of peak picking and alignment. Peak pick on any run were edited and the same change to the same feature was made across all runs.

Once a list of detected compound ions was identified, Progenesis called the PLGS search algorithm (Waters Corporation) to perform database searching based on accurate peptide mass and fragmentation data. The database for *Streptoccocus sanguinis* was downloaded from Uniprot. For easier management of large amounts of data and sample comparison, we used Scaffold Q+ software (http://www.proteomesoftware.com/products/qplus/)

## 3. Results and Discussion

**Figure 1:**
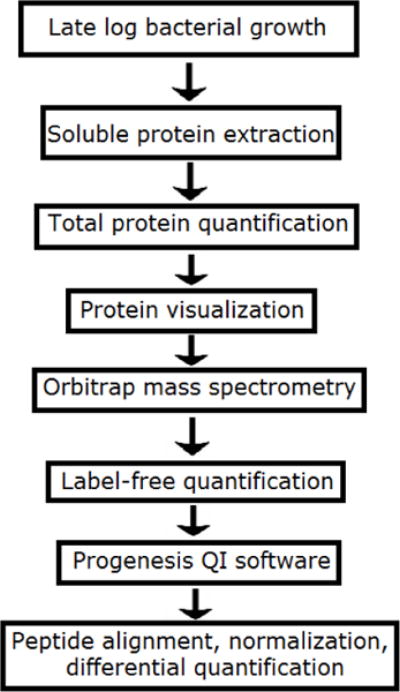
Schematic presentation of the multi-step protocol for high resolution soluble proteome analysis

An essential biological aim in bacteriology is the identification of protein dynamics in bacteria during different physiological phenomena, complementing the transcriptomics, metabolomics, and genomics data to provide deep insights into bacterial behavior. *S. sanguinis* SK36 is Janus-faced microorganism: an oral commensal and opportunistic etiologic agent of infective endocarditis that our lab has sequenced its genome (Xu et al., 2007) and identified its set of essential genes (Xu et al., 2011), creating a library of mutants that can provide invaluable information about the impact of every knocked out gene on the bacterial homeostasis. The current challenge was to identify a proteomics pipeline for extraction, quantification, and differential analysis of *S. sanguinis* SK36 proteins, hopefully to define “pathogenesis signatures” as promising therapeutic targets (Figure 1).

Despite the rapid development pace of mass spectrometry technology, accompanied by high accuracy and comprehensive coverage of proteins, implementing quantitative proteomics workflows has been limited in Gram-positive bacterial model (Herbst, Siebourg, Mäder, Lalk, Hecker, 2010; Becher, Meyer, Lalk, Schurmann, Schlüter, 2012; Muntel, Hecker, Becher, 2012; Pierce, Rees, Fernandez, Barr, 2012; Rivera, Miller, Kolar, Stevens, Shaw, 2012; Chapman, Balasubramanian, Tam, Askenazi, Copin, 2017). This has been attributed to many reasons, such as presence of a thick cell that is resistant to conventional cell lysis procedures, limited availability of commercial ready-to-use kits such as Qproteome Bacterial Protein Prep Kit (Qiagen), CelLytic B Plus (Sigma-Aldrich) and B-PER™ with Enzymes Bacterial Protein Extraction Kit (Thermo Scientific), species-to-species proteomic variations which demand the use of lysostaphin for *Staphylococcus aureus* and mutanolysin for *Streptococcus mutans*, and low protein yield due to harsh protein extraction methods (Santoni, Molloy, & Rabilloud, 2000; Rabilloud, 2009).

Protein extraction represents the bottleneck step in any proteomics pipeline as it impacts all following steps and requires a lot of knowledge and technical expertise in handling samples at optimal conditions. A major dilemma in protein extraction procedures is the use of EDTA: it inhibits metalloproteases that are abundant in many bacterial proteomes, but unfortunately, EDTA inhibits DNase activity which is required to reduce the lysate viscosity and prevent the sedimentation of the extracted proteins under the precipitating genomic DNA that is so large and abundant that as it precipitates it can trap proteins or even organelles. According to NCBI annotations, *S. sanguinis* SK36 strain has 12 metalloproteases and 5 additional putative metalloproteases, which necessitates the need for metalloprotease inhibitor as EDTA. The use of sonication as a mechanical method has been advised for its high efficiency in cell wall disruption in other bacterial models (Schwarz, Fiedler, Fischer, Bahl, 2007; Sianglum, Srimanote, Wonglumsom, Kittiniyom &Voravuthikunchai, 2011) and for bypassing the need to use DNase as sonication is a DNA-fragmenting technique as well (Table 1). Sonication stands out as the physical method of choice for disruption of bacterial cell walls, as the other major option, French press, has many limitations that reduce its feasibility: sample volume should be more than 100 ml, loss of proteins from the heat elicited by the pressing machine, technically demanding machine that is not abundant in many research facilities.

**Figure 2:**
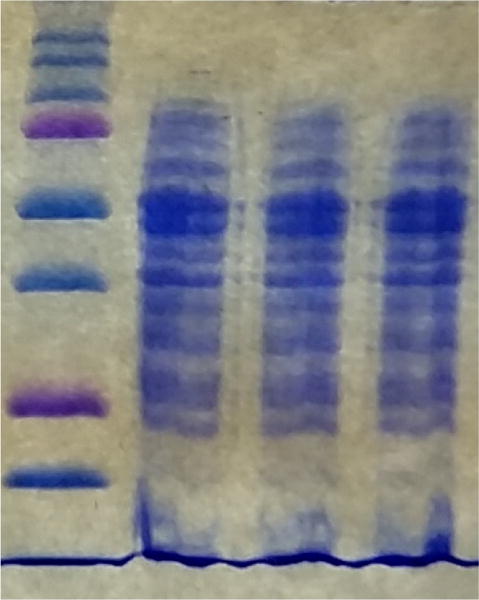
Coomassie Blue stained gel of soluble proteins extracted from *Streptococcus sanguinis* SK36. (from left) Lane 1: Protein ladder; Lanes 2–4: soluble proteins extracted from *S. sanguinis* SK36 in triplicate

Another major obstacle in designing protein extraction procedures is the interference of chemicals used at different stages that may affect downstream applications, such as protein quantification and mass spectrometry analysis. Many conventionally used chemicals for protein extraction have been shown to induce lateral damage. Glycerol which is used for gradient sedimentation has been shown to clog mass spectrometry channels (Kress, Meissner, Kaiser, Wood, 2002). Conventional detergents like CHAPS, β-mercaptoethanol, and NP-40 had to be replaced by DTT and 1% SDS to reduce complications in mass spectrometry. In addition, many protein quantification kits provide a list of chemicals that should be avoided to prevent false readings of results. The Pierce BCA Protein Assay Kit that we used has a list of permissive doses of buffers, detergents, chelating and reducing agents that can be used, limiting our options even further. The extraction method that we have used relies on the minimal use of chemical agents in lowest permissive doses, providing a feasible approach that can be safely used for multiple kits and provide highest specificity and sensitivity (Table 1). To confirm the abundance of proteins in samples and avoid misreadings of protein quantification kits, we added a Coomassie stainig step (Figure 2) to the pipeline in order to visualize the proteins as bands before moving to downstream steps that prepare the samples for mass spectrometry analysis.

Label-free mass spectrometry-based proteomic approaches are fundamental tools for deciphering bacterial proteome diversity and dynamics, given a surfeit of advantages such as financial efficiency in comparison to isotope labelled techniques, technical feasibility for handling many samples, wide application on various experimental settings including cases when isotope-labelling approach is less favored, such as non-cultivatable bacteria or slow-growing bacterial scenarios, such as stressed bacteria or biofilm-inhabiting bacteria (Griffin, et al., 2010; Li, et al., 2012; Porteus, Kocharunchitt, Nilsson, Ross, Bowman, 2011; Hettich, Pan, Chourey, Giannone, 2013). Tandem mass spectra were extracted and all MS/MS samples were analyzed using ProteinLynx Global Server (Waters Corporation, Milford, MA; version 3.0.3). ProteinLynx Global Server was set up to search the uniprot_streptococcus_sanguinis_160527.fasta_def database. ProteinLynx Global Server was searched with a fragment ion mass tolerance of 1.00 PPM and a parent ion tolerance of 1.00 PPM. Carbamidomethyl of cysteine was specified in ProteinLynx Global Server as a fixed modification. Oxidation of methionine was specified in ProteinLynx Global Server as a variable modification. The proteome of *S. sanguinis* SK36 was investigated previously and the coverage was 28.4% (Xu et al., 2007), but with current protocol, we achieved a coverage of soluble proteins (Protein probability score cutoff >5%) equivalent to 58.8%, 53.2%, 61.6% from treated samples 1, 2, and 3 respectively. The current coverage is double the previous coverage, and yet with further refinement and technological advances, this coverage is expected to increase.

Progenesis QI software was used to help us put our results into a wider biological context. Quantification data, including raw abundance of every isotope peak for every adduct, and identification results were easily exported and in future will be linked with other ‘omics data or used as the basis for further bioinformatics.

**Figure 3:**
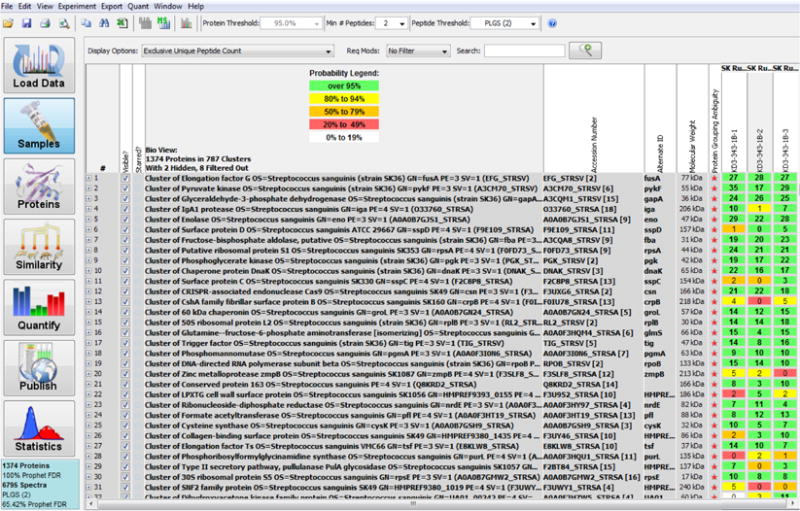
The peptides and proteins identified for the samples (soluble proteins) are shown in the Scaffold file. Many proteins from Streptococcus were identified as upregulated (green). From right, the three lanes (1–3) are displays of protein from wild type *S. sanguinis* strain run in triplicate

Scaffold (version Scaffold_4.7.2, Proteome Software Inc., Portland, OR) was used to validate MS/MS based peptide and protein identifications. Peptide identifications were accepted if they exceeded specific database search engine thresholds. Protein identifications were accepted if they contained at least 2 identified peptides. Proteins that contained similar peptides and could not be differentiated based on MS/MS analysis alone were grouped to satisfy the principles of parsimony. A total of 1374 proteins sharing significant peptide evidence were grouped into 787 clusters (Figure 3).

The current protocol will pave the way for tackling emerging hot topics in oral bacteria proteomics, such as *in vivo* proteomics as related to host-bacterial interaction, biofilm formation, dental infections, infective endocarditis, and potentially combine data with transcriptomic, genomic, and metabolomic findings for a systems biological modeling approaches.

## Figures and Tables

**Table 1:** Protein extraction data from *S. sanguinis* SK36 strain in triplicate

Sample	Treatment	DNA concentration* (μg/mL)	Protein concentration (ng/μL)	Number identified protein by mass spectrometryѱ
Treated sample A	Sonication	958	2516.6	937
Treated sample B	Sonication	1007	2626.2	948
Treated sample C	Sonication	1089	2874.2	981
Untreated sample A	–	315	216.4	ND
Untreated sample B	–	338	257.3	ND
Untreated sample C	–	323	206.6	ND

* DNA concentration was measured directly after sonication to detect efficiency of sonication in cell wall disruption.

ѱ Total count of putative soluble proteins is 1593.

ND = not determined.
